# Uncovering the similarities of lipidome-wide markers of carotid artery plaque and metabolic dysfunction-associated fatty liver disease: the Young Finns study

**DOI:** 10.1038/s41598-026-51430-0

**Published:** 2026-05-06

**Authors:** Haniyeh Danest Doost, Terho Lehtimäki, Reija Autio, Juhani S. Koskinen, Reijo Laaksonen, Nina Mononen, Mika Kähönen, Olli Raitakari, Juha Mykkänen, Katja Pahkala, Suvi P. Rovio, Binisha H. Mishra, Pashupati P. Mishra

**Affiliations:** 1 Department of Clinical Chemistry, Fimlab Laboratories, and Finnish Cardiovascular Research Center - Tampere, Faculty of Medicine and Health Technology, Tampere University, Tampere, Finland; 2 Faculty of Social Sciences (Health Sciences), Tampere University, Tampere, Finland; 3 Research Centre of Applied and Preventive Cardiovascular Medicine, University of Turku, Turku, Finland; 4 Centre for Population Health Research, Turku University Hospital, University of Turku, Turku, Finland; 5 Department of Medicine, University of Turku, Division of Medicine, Turku University Hospital, Turku, Finland; 6 Department of Medicine, Satakunta Central Hospital, Pori, Finland; 7 Department of Clinical Chemistry and Finnish Cardiovascular Research Center - Tampere, Faculty of Medicine and Health Technology, Tampere University, Tampere, Finland; 8 Zora Biosciences Oy, Espoo, Finland; 9 Department of Clinical Physiology, Tampere University Hospital, and Finnish Cardiovascular Research Center - Tampere, Faculty of Medicine and Health Technology, Tampere University, Tampere, Finland; 10 Department of Clinical Physiology and Nuclear Medicine, Turku University Hospital, Turku, Finland; 11 InFLAMES Research Flagship, University of Turku, Turku, Finland; 12 Tampere University Hospital, Wellbeing Services County of Pirkanmaa, Tampere, Finland

**Keywords:** Lipidomics, Cardiovascular disease, Fatty liver, Carotid plaque, Phosphatidylcholine, Biomarkers, Cardiology, Diseases, Medical research

## Abstract

Metabolic dysfunction-associated fatty liver disease (MAFLD) and carotid artery plaque (CAP) are both linked to circulatory lipid and lipoprotein metabolism. However, the shared lipidome-wide mechanisms underlying these diseases remain unexplored. To identify plasma lipid species associated with both MAFLD and CAP to uncover their shared metabolic pathways. We analyzed data from the Young Finns Study cohort from the 2007 and 2018 follow-ups (n = 1496, aged 41–56 years, 56.3% females). Ultrasound was used to determine the prevalence of both CAP and MAFLD during the 2018 follow-up. The participants were categorized into three mutually exclusive groups: participants with CAP without MAFLD (n = 257), participants with MAFLD without CAP (n = 150), and a control group free from both diseases (n = 436). Lipidomic profiling of 437 lipid species from plasma was performed during the 2007 follow-up (aged 30–45 years) via liquid chromatography‒tandem mass spectrometry. Logistic regression models, both unadjusted and adjusted for age, sex, physical activity, alcohol consumption, and smoking, were used to assess lipid associations with both disease outcomes separately. Odds ratios (ORs) and confidence intervals (95% CIs) were calculated for each lipid species, and multiple testing corrections were performed via the false discovery rate (FDR) method (< 0.05). Additionally, we performed a hypergeometric enrichment analysis to determine whether certain lipid classes appear more often than expected among the lipids associated with disease. In the unadjusted models, there were a total of 51 significant (FDR < 0.05) overlapping lipids between the CAP and MAFLD groups. In the adjusted models, four lipids were significantly associated with CAP, and 202 lipids were significantly associated with MAFLD. Notably, only one lipid—phosphatidylcholine (PC) 40:4—was significantly associated with both diseases. PC 40:4 was associated with an increased risk of CAP (OR 2.59; 95% CI, 1.57–4.32) and MAFLD (OR 5.26; 95% CI, 2.81–9.85). Our findings highlight PC 40:4 as a novel shared lipid signature for both MAFLD and CAP. This dual association suggests that overlapping metabolic disturbances and potentially common lipid-based pathogenic mechanisms link liver and vascular health. PC 40:4 may serve as a promising early biomarker or therapeutic target for metabolic-vascular comorbidities.

**Supplementary Information:**

The online version contains supplementary material available at 10.1038/s41598-026-51430-0.

## Introduction

Cardiovascular diseases (CVDs) remain among the leading causes of death globally and are strongly linked to serious cardiometabolic comorbidities^1^. Among these comorbidities, metabolic dysfunction-associated fatty liver disease (MAFLD) and atherosclerosis are increasingly recognized as interconnected conditions that share a common metabolic origin. Both are complex, multifactorial diseases driven by overlapping risk factors—including insulin resistance, dyslipidemia, obesity, and chronic inflammation—and often cooccur within the context of metabolic syndrome^2–4^.

Atherosclerosis progresses silently from early life into adulthood, potentially affecting various vascular systems^5^. Carotid artery plaque (CAP), in particular, has emerged as a strong predictor of cardiovascular events and mortality^6^. Several studies have shown that individuals with MAFLD are more likely to develop signs of subclinical atherosclerosis, such as increased carotid intima‒media thickness and plaque, even after accounting for traditional risk factors^7–9^. The accumulation of fat in the liver may lead to changes in lipoproteins and promote inflammatory responses that affect blood vessels. Moreover, long-term exposure to an unhealthy lipid profile may contribute to both liver damage and vascular disease^10,11^. Despite increasing recognition of their co-occurrence, the molecular pathways linking MAFLD and atherosclerosis are still not fully understood.

Lipidomics, the comprehensive profiling of lipid species via high-resolution mass spectrometry, is a powerful tool for investigating these mechanisms^12^. Unlike traditional lipid panels, lipidomics can quantify hundreds of lipid molecules simultaneously, revealing detailed metabolic fingerprints associated with disease. This approach has enhanced our understanding of lipid biology and holds promise for identifying novel biomarkers and therapeutic targets^13,14^. However, while many studies have examined the lipidomic signatures of either MAFLD or atherosclerosis individually, there remains a significant gap in the literature regarding studies that search for shared lipid profiles between these diseases. Exploring common molecular lipid signatures may provide insights into overlapping mechanisms driving both diseases and could reveal novel targets for integrated diagnostic and therapeutic approaches.

To address this gap, we aimed to identify shared and disease-specific plasma lipid species associated with MAFLD and CAP via lipidomic data from the Young Finns Study, one of the largest longitudinal studies on cardiovascular risk from childhood to adulthood. We hypothesized that these conditions (MAFLD and atherosclerosis) exhibit overlapping lipidomic signatures reflecting common metabolic pathways, alongside distinct disease-specific lipid profiles. By analyzing overlapping lipid signatures, we sought to enhance our understanding of lipid metabolism and its potential role in the development of CVD and its comorbidities. These findings could offer new insights into the pathophysiology of cardiometabolic disease and guide future strategies for risk assessment and prevention.

## Subjects and methods

### Study population and ethics

The Young Finns Study (YFS) is a Finnish population-based, multicenter prospective longitudinal study examining the progression of cardiovascular risk factors from childhood to adulthood^15^. The study was initiated in 1980 with a baseline cohort of 3596 children and adolescents (aged 3–18 years) randomly selected from the national population registry across five university hospital regions in Finland (Turku, Tampere, Helsinki, Kuopio, and Oulu). For this study, we used YFS lipidomic data measured at the 2007 follow-up and MAFLD and CAP data measured 11 years later in the 2018 follow-up. To assess the potential presence of early-stage disease prior to 2018, we examined earlier imaging data available in the YFS cohort. Carotid plaque assessments were conducted in 2007, and liver steatosis was evaluated in 2011.

Out of the 3596 participants in YFS baseline, 1496 had complete lipidomic and phenotypic data and were eligible for analysis. Among them, 653 participants presented with both MAFLD and CAP in 2018 and were excluded from the primary comparison to ensure mutually exclusive disease groups. This exclusion was necessary to enable disease-specific interpretation and prevent confounding due to overlapping pathologies. The remaining 843 participants were categorized into three groups: those with CAP but without MAFLD (n = 257), those with MAFLD but without CAP (n = 150), and a control group without either condition (n = 436). Each disease group was compared separately to the common control group. The control group represents metabolically healthier individuals, free of both liver and vascular pathology as assessed in 2018 and served as a common reference for comparison. A flow diagram showing participant selection for the analysis is provided in Figure S1. A total of 437 lipid profiles were analyzed from 2007 serum samples (see methods).

Informed consent was obtained from all participants or their legal guardians. The study protocol was approved by the Ethics Committee of the Hospital District of Southwest Finland (ETMK:68/1801/2017). All methods were performed in accordance with the relevant guidelines and regulations.

### Health and lifestyle characteristics

Health and lifestyle information was collected during the 2018 follow-up. Physical activity was assessed via a self-administered questionnaire that captured details on the frequency, intensity, and duration of both leisure-time activities and commuting to work. The metabolic equivalent (MET) index, expressed in MET hours per week (MET h/wk), was computed by multiplying intensity, frequency, and duration, encompassing both leisure-time and commuting activities^16,17^. One MET corresponds to the energy expenditure of one kilocalorie per kilogram of body weight per hour at rest. The MET index also incorporates commuting activity, taking into account the mode of transportation and seasonal variations^17^. Alcohol consumption was assessed through a questionnaire in which participants reported their intake of beer, wine, and liquor over the past week, with total consumption calculated in units of alcohol (14 g per unit)^18^. Smoking status was assessed during a medical examination in a private room to ensure that participants could respond confidentially and without interruption. Regular smoking was defined as daily cigarette use during adolescence and adulthood^19^. Data on body mass index (BMI), diabetes, and hypertension were available, but we did not include BMI, diabetes, or hypertension as covariates in the main models. These factors are strongly interrelated with lipid metabolism and disease outcomes, and excluding them avoids potential overadjustment since they may lie on the causal pathway.

### Measurement of CAP

Carotid ultrasound studies were performed via GE Logiq S8 mainframes (GE Vingmed Ultrasound A/S, Horten, Norway) equipped with an ML6-15-D matrix linear transducer. Standardized protocols were followed by sonographers and trained ultrasound technicians to assess CAP in the left and right common carotid artery, carotid bifurcation, and internal carotid artery^20^. CAP was defined as a focal structure protruding into the arterial lumen by at least 0.5 mm, by 50% of the surrounding IMT value, or having a thickness > 1.5 mm measured from the media–adventitia interface to the intima–lumen interface^21^. All the scans were stored for offline analysis, and CAP measurements were performed by one experienced reader blinded to the participants’ details. CAP was treated as a binary variable, where the presence of a plaque was coded as 1 and absence as 0. A score of 1 was assigned if the participant had at least one plaque visible in the image.

### Measurement of MAFLD

Hepatic ultrasound imaging to assess liver fat content was performed via a Logiq S8 (GE Vingmed Ultrasound A/S, Horten, Norway) ultrasound device with a 1.5–6.0 MHz convex C1–6 transducer, following a validated protocol. The estimation of hepatic steatosis was based on liver-to-kidney contrast, parenchymal brightness, deep beam attenuation, bright vessel walls, and visibility of the neck of the gallbladder. A single trained sonographer, blinded to the participants’ clinical characteristics, visually evaluated the images and classified the participants as having a fatty liver or normal liver.

### Lipidome-wide analysis

A total of 437 lipid profiles were analyzed, representing five lipid classes, such as fatty acyls, glycerolipids, glycerophospholipids, sphingolipids, and sterol lipids, and 15 lipid subclasses, including acylcarnitine, cholesteryl ester (CE), ceramide (Cer), diacylglycerol (DAG), globoside (Gb3), glycosylceramide (Glc), lactosylceramide (LacCer), lysophosphatidylcholine (LPC), lysophosphatidylethanolamine (LPE), phosphatidylcholine (PC), phosphatidylethanolamine (PE), phosphatidylglycerol (PG), phosphatidylinositol (PI), sphingomyelin (SM), and triacylglycerol (TAG). Lipidomic analysis of the serum samples was carried out at Zora Biosciences Oy (Espoo, Finland) via an extraction protocol from previously published techniques^22^. Briefly, 10 µL of serum was mixed with 10 µL of 10 mM 2,6-di-tert-butyl-4-methylphenol (BHT) in methanol to prevent oxidation. Next, 20 µL of class-specific internal lipid standards (Avanti Polar Lipids Inc., Alabaster, AL) and 300 µL of a chloroform:methanol (2:1, v/v) mixture (Sigma‒Aldrich) were added. The mixture was vortexed, sonicated for 10 min and incubated for 40 min. After centrifugation at 5700 × *g* for 15 min, the upper organic phase was collected, evaporated under nitrogen gas, and resuspended in 100 µL of water-saturated butanol. The reconstituted solution underwent an additional sonication step (5 min) before the addition of 100 µL of methanol. A final centrifugation at 3500 × *g* for 5 min was performed to remove particulates, and the supernatant was used for MS analysis^23^. Lipid profiling was conducted via a hybrid triple quadrupole/linear ion trap mass spectrometer (QTRAP 5500, AB Sciex, Concord, Canada) coupled with a Nexera-X2 ultrahigh-performance liquid chromatography (UHPLC) system (Shimadzu, Kyoto, Japan). Identification of diacyl lipids such as phosphatidylcholines was performed at the species level, defined by the total number of carbon atoms and double bonds. This sum-composition approach is standard in multiple reaction monitoring (MRM)-based lipidomics and allows robust quantification, though it does not resolve isomers (e.g., sn-position, double-bond location). Lipids were separated on an Acquity BEH C18 column (2.1 × 50 mm, 1.7 µm; Waters Corporation, Milford, MA, USA). Only lipids detected in ≥ 90% of the samples were retained to minimize bias from missing data. Quality control was ensured through the inclusion of pooled plasma samples and repeated injections, which enabled batch-level performance monitoring and alignment. Analytical variability was assessed via intrabatch coefficients of variation, which were generally < 15% for major lipid classes, supporting the reproducibility of the platform. Data acquisition was performed via a scheduled MRM algorithm, and peak intensities were normalized to the appropriate class-specific internal standards via Analyst and MultiQuant 3.0 software (AB Sciex)^24^.

### Statistical methods

Descriptive analyses were conducted to summarize participant characteristics. Categorical variables are reported as counts (n) and percentages (%), whereas continuous variables are presented as the means and standard deviations (SD). The proportions of various conditions, such as diabetes, obesity, and hypertension, were assessed via frequency tables. The lipid profiles were log-transformed to correct skewness. To assess the associations between lipid species and disease outcomes, logistic regression models were applied separately for each lipid species. Both unadjusted and adjusted models were utilized. The adjusted models accounted for covariates from 2018, including continuous variables (age, physical activity, alcohol consumption) and categorical variables (sex, smoking status). Importantly, we did not adjust for BMI, diabetes, or hypertension in our primary models, as these factors might actually be part of the process linking lipids to disease. Including them could introduce overadjustment bias and obscure true biological associations. For each lipid model, odds ratios (ORs), 95% confidence intervals (CIs), and *p* values were reported. To account for multiple testing, we applied the Benjamini–Hochberg false discovery rate (FDR) correction separately within each analysis. That is, *p* values for all 437 lipids were adjusted to control the FDR for the CAP analysis and independently for the MAFLD analysis. Lipid associations with an FDR < 0.05 were considered statistically significant. Finally, to identify enriched lipid classes among significant species, we performed a hypergeometric test for overrepresentation of subclasses.

All the statistical analyses and data processing were conducted via R version 4.3.1. with the ComplexHeatmap^25^ and VennDiagram^26^ packages.

## Results

### Study population characteristics

The baseline characteristics of the participants are presented in Table 1.

**Table 1:** Population characteristics associated with cardiovascular risk in the Young Finns study cohort. The data are presented as the means ± SDs or percentages.

	MAFLD Cases (n = 150)		CAP Cases (n = 257)		Controls (n = 436)	
	Women	Men	Women	Men	Women	Men
Age (years)	49.6 ± 4.7	48.1 ± 4.9	50.5 ± 4.7	49.9 ± 4.6	48 ± 4.7	46.9 ± 5
Physical activity (MET-hours/week)	12.7 ± 17.7	17.6 ± 17.1	18.1 ± 15.8	22.7 ± 19.7	21.5 ± 18.7	23 ± 20.7
Alcohol consumption (units/day)	0.5 ± 0.6	0.9 ± 1	0.4 ± 0.4	0.8 ± 1.1	0.4 ± 0.6	0.9 ± 0.9
Smoking (%)	5/75 (6.7%)	10/75 (13.3%)	12/151 (7.9%)	17/106 (16%)	28/295 (9.5%)	24/141 (1.7%)
Participants with diabetes (%)	11/75 (14.7%)	9/75 (12%)	5/151 (3.3%)	5/106 (4.7%)	7/295 (2.4%)	2/141 (1.4%)
Participants with obesity (%)	50/75 (66.7%)	39/75 (52%)	26/151 (17.2%)	17/106 (16%)	46/295 (15.6%)	15/141 (10.6%)
Participants with hypertension (%)	23/75 (30.7%)	19/75 (25.3%)	23/151 (15.2%)	21/106 (19.8%)	23/295 (7.8%)	12/141 (8.5%)

Metabolic dysfunction-associated fatty liver disease, MAFLD; carotid artery plaque, CAP.

### Assessment of pre-existing disease prior to 2018

To assess potential pre-existing disease, earlier phenotype data were examined within the analyzed CAP-only and MAFLD-only groups. Among the 257 CAP cases, all had plaque data available from 2007, and 15 (5.8%) already had plaque at that earlier time point. Among the 150 MAFLD cases, 137 had fatty liver data available from 2011, and 47 of these (34.3%) already had liver fat accumulation.

### Associations between lipid profiles and CAP

In the adjusted models, we identified four lipid species, three PCs and one Cer, which were significantly (FDR < 0.05) associated with CAP risk. All of these factors showed strong positive associations and increased risk (OR > 1 for all) (Table S1) with CAP. In the adjusted model, all lipid species significantly associated with CAP are shown in Figure S2 (panel A). To assess potential overrepresentation of lipid subclasses among the CAP-associated lipids, we applied a hypergeometric enrichment analysis. PCs had an enrichment ratio of 3.23, and Cers had a ratio of 3.70, indicating that these lipid subclasses appeared more than three times as frequently among the significant lipids than expected by chance. However, these enrichments did not reach statistical significance after correction for multiple testing (PC: FDR = 0.618; Cer: FDR = 1.00). This result shows that although individual PCs and Cers are significantly associated with CAP, there is no strong evidence that these classes as a whole are systematically enriched for CAP-related alterations. The full results of the enrichment analysis are provided in Table S2.

In addition, unadjusted models identified 80 lipid species associated with CAP in 12 lipid subgroups, all of which were positively associated with increased risk, as detailed in Table S3 and shown in Figure S3.

### Associations between lipid profiles and MAFLD

**Fig. 1:**
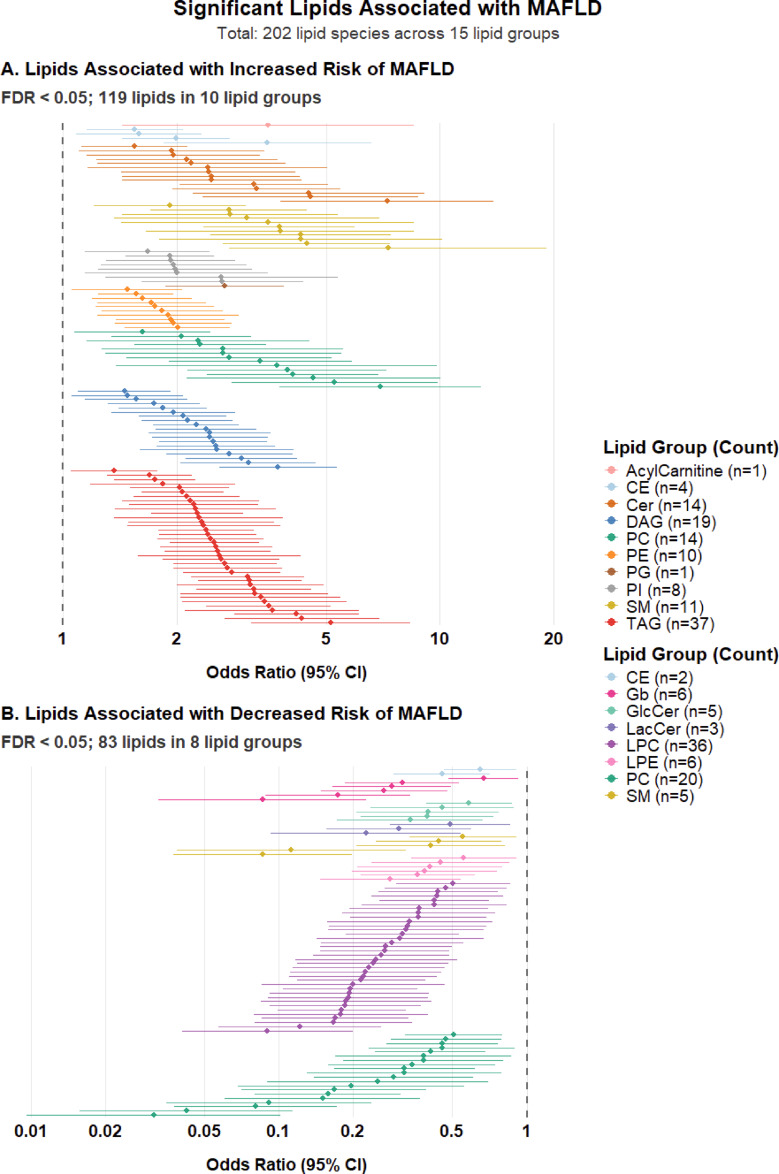
Lipid species associated with MAFLD with an FDR < 0.05. Forest plot showing odds ratios (ORs) and 95% confidence intervals (CIs) for 202 lipid species significantly associated with MAFLD risk (FDR < 0.05). Panel (**A**) presents 119 lipids from 10 lipid groups associated with increased risk. Panel (**B**) shows that 83 lipids from 8 lipid groups are associated with decreased risk. The lipid groups are abbreviated as follows: TAG, triacylglycerol; DAG, diacylglycerol; PC, phosphatidylcholine; LPC, lysophosphatidylcholine; PE, phosphatidylethanolamine; LPE, lysophosphatidylethanolamine; PG, phosphatidylglycerol; PI, phosphatidylinositol; SM, sphingomyelin; Cer, ceramide; LacCer, lactosylceramide; GlcCer, glucosylceramide/galactosylceramide; Gb, globotriaosylceramide; CE, cholesteryl ester; Acylcarnitine, acylcarnitine.

A total of 202 lipid species were significantly associated with MAFLD risk according to the adjusted models (FDR < 0.05), as shown in Table S4. Significant lipid associations with MAFLD are presented in Fig. 1, highlighting lipids linked either to increased or decreased risk. Moreover, Figure S2 (panel B) comprehensively presents all lipid species significantly associated with MAFLD risk in the adjusted model. Among these lipids, 119 lipids were associated with increased risk (OR > 1 for all), including 37 TAGs, 19 DAGs, 14 PCs, 14 Cers, 11 SMs, 10 PEs, 8 PIs, 4 CEs, 1 PG, and 1 acylcarnitine. Conversely, 83 lipid species were associated with a decreased risk of MAFLD (OR < 1 for all), including 36 LPCs, 20 PCs, 6 LPEs, 6 Gbs, 5 GlcCers, 5 SMs, 3 LacCers, and 2 CEs. The hypergeometric enrichment analysis revealed significant enrichment for several lipid subclasses. The analysis identified TAGs and DAGs as highly enriched among the significantly associated lipids. Specifically, 37 out of 42 TAGs and all 19 DAGs were significantly associated with MAFLD, with enrichment ratios of 1.91 and 2.16 and FDRs of 5.49 × 10⁻⁸ and 2.00 × 10⁻⁶, respectively. Additionally, LPCs and Gbs were significantly enriched, although at a lower magnitude (FDR = 0.023 and 0.035, respectively). Other lipid subclasses, including Cers, SMs, and PCs, did not demonstrate statistically significant enrichment (FDR > 0.05). The full enrichment results are presented in Table S5. These findings indicate that TAGs and DAGs are particularly overrepresented among MAFLD-associated lipids, suggesting their potential involvement in the underlying metabolic alterations in MAFLD. In contrast to CAP, MAFLD does exhibit subclass-specific lipid enrichment within the detected lipidome.

According to the unadjusted models, 218 lipid species were associated with MAFLD, including 134 with increased risk and 84 with decreased risk (Table S6), the details are shown in Figure S4.

### Lipid species associated with both MAFLD and CAP

**Fig. 2:**
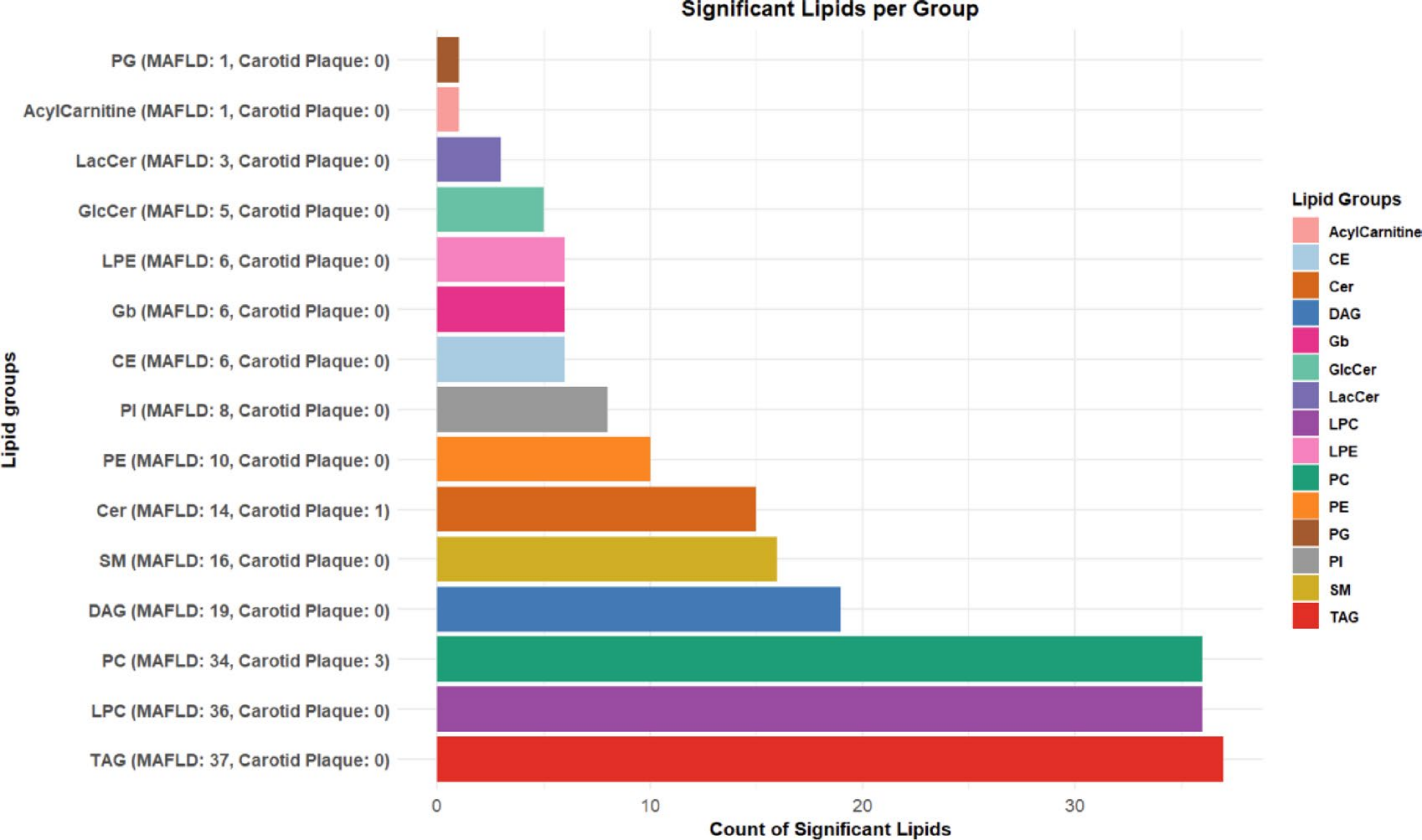
Distribution of significant lipids by lipid class in association with MAFLD and CAP. This bar chart displays the count of significant lipid species (FDR < 0.05) per lipid subclass, with numbers in parentheses indicating the count of significant lipids for MAFLD and CAP (format: MAFLD: count, CAP: count).

**Fig. 3:**
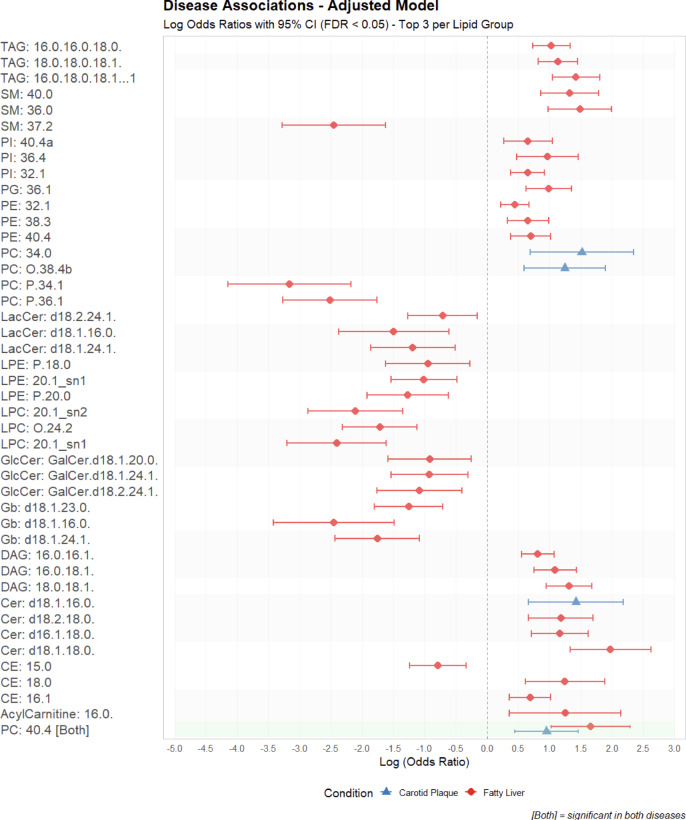
Forest plots illustrating the associations between lipid species and two diseases: CAP and MAFLD. The results are expressed as log odds ratios with 95% confidence intervals (CIs) for lipids significantly associated with each disease (FDR < 0.05). The panel shows the adjusted model, accounting for covariates such as age, sex, physical activity, alcohol consumption, and smoking status. The top 3 lipids per lipid group are highlighted on the basis of the minimum FDRs. In addition, the most significant overlapping lipids per class (based on FDR) are included to highlight shared associations between the two diseases, and these overlapping lipids are marked as “[Both]” in the adjusted model. Red points and lines represent associations with fatty liver, blue points and lines represent associations with carotid plaque, and green shading highlights lipids that are significant in both diseases. *Note: Due to the wide range of odds ratios across lipid species, log-transformed odds ratios are used to symmetrize the scale around zero, simplifying the visualization and comparison of both the direction and magnitude of associations.

**Fig. 4:**
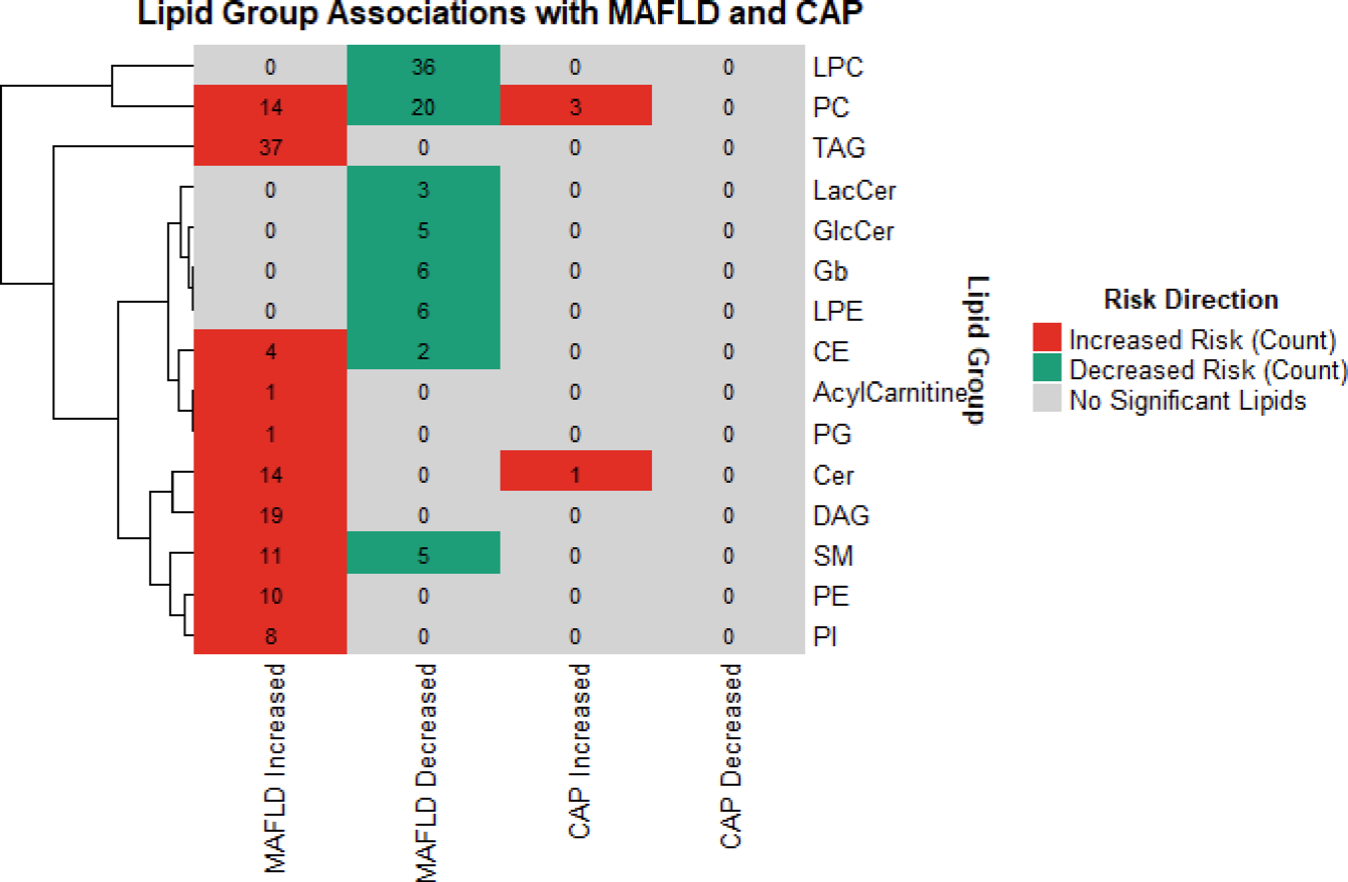
Heatmap of associations between lipid groups and disease risk for MAFLD and CAP. Each cell shows the number of lipids within a group that were significant in logistic models at FDR < 0.05, stratified by risk direction (red = increased risk; green = decreased risk; gray = not significant). Rows (lipid groups) are ordered by hierarchical clustering of the rowwise count profiles via Euclidean distance and complete linkage. Counts are printed inside cells.

Several lipid subclasses, including PCs, LPCs, DAGs, TAGs, and Cers, emerged as being jointly associated with the studied diseases. The distributions of all significant lipid species by lipid subclass are shown separately for MAFLD and CAP in Fig. 2. The three most significant lipids per lipid subclass, selected on the basis of the lowest FDR values for each of the diseases (MAFLD and CAP) in the adjusted models, are shown in Fig. 3; similarly, the unadjusted models are shown in Figure S5. According to the adjusted model, only one lipid species out of 437 lipids was significantly associated with both MAFLD and CAP. This lipid, belonging to the phosphatidylcholine group (PC 40:4), was associated with an increased risk under both conditions. The ORs were 5.26 (95% CI, 2.81–9.85) for MAFLD and 2.59 (95% CI, 1.57–4.32) for CAP. This finding may reflect a potential shared metabolic pathway underlying both conditions, despite their nonoverlapping occurrence in the study population. Our study identified 51 shared and statistically significant lipid species via unadjusted models, 23.4% of which were lipids identified in MAFLD and 63.8% of which were lipids identified in CAP (Figure S6). A summary of the shared lipid species associated with CAP and MAFLD in nonoverlapping participant populations is shown in Table S7. The large number of lipid associations in unadjusted models decreased substantially after covariate adjustment and FDR correction were applied, emphasizing how these steps are essential for reducing false positives and isolating the most reliable lipid–disease links. A detailed breakdown of these associations is available in Table S8. The distribution of lipid species on the basis of their associations with different numbers of diseases is as follows: 232 lipids showed no association with any studied disease, 204 lipids were associated with at least one disease, and only 1 lipid (PC 40:4) demonstrated significant associations with two diseases. The number of lipid species within each lipid class that are significantly associated with MAFLD and CAP is shown in Fig. 4, distinguishing between those linked to increased or decreased disease risk.

## Discussion

To our knowledge, no previous study has examined the overlapping lipidomic features of MAFLD and CAP within the same population cohort using non-overlapping disease groups. This approach enabled a clear comparison of lipids linked exclusively to each condition, as well as those common to both. Among 437 lipid species profiles, PC 40:4 was the only lipid significantly associated with both MAFLD and CAP after adjustment for key covariates. This overlap points to potential metabolic pathways shared by liver steatosis and atherosclerosis.

Although unadjusted models identified multiple overlapping lipid associations, most lost statistical significance after covariate adjustment, emphasizing the influence of confounding factors. The persistent association of PC 40:4 in fully adjusted models highlights its potential relevance as a robust marker of cardiometabolic dysregulation.

While our lipidomic data were measured only at a single time point in 2007 and there was an 11-year interval between lipidomics profiling and clinical phenotyping, previous studies have shown that many plasma lipids remain stable over time and can still predict cardiometabolic diseases years later^27,28^. For example, in the Young Finns study itself, Würtz et al. reported that circulating lipid and metabolite profiles measured at baseline predicted cardiovascular outcomes over a decade of follow-up^29^. Similarly, several plasma lipid species, including PCs, maintained moderate-to-high intraclass correlation coefficients over a 10-year period.

PC 40:4 was identified at the lipid species level based on its sum composition—i.e., the total number of carbon atoms and double bonds—without resolution of structural isomers such as acyl chain positions or configurations. As such, the measured signal may reflect a group of isomeric phosphatidylcholine species rather than a single defined molecular entity. Its consistent association with both MAFLD and CAP points to potential overlapping metabolic pathways linking hepatic and vascular disease processes.

Although prior studies support the long-term stability of many plasma lipid classes, it is important to note that PC 40:4 is a polyunsaturated species and potentially susceptible to oxidative degradation during storage. However, all samples in this study were stored under standardized, low-temperature conditions with consistent quality control protocols were applied across the cohort. Therefore, while the possibility of selective degradation cannot be entirely excluded, it is unlikely to account for the observed disease-specific associations. Therefore, these findings support the biological plausibility of early lipidomic signatures, including PC 40:4, as stable indicators of future cardiometabolic diseases.

To better understand disease-specific lipid involvement, we also performed enrichment analyses. TAGs and DAGs were overrepresented among lipids associated with MAFLD. These lipid classes are functionally relevant: TAGs are involved in lipid storage, whereas DAGs serve as intermediates in lipid signaling and metabolism^30,31^. In addition, Cers, which contribute to insulin resistance, apoptosis, and inflammatory signaling, were enriched among both MAFLD- and CAP-associated lipids^32,33^. Although the PC class as a whole was not enriched, the consistent association of PC 40:4 suggests that particular PC molecules are critical in the common pathobiology of CAP and MAFLD. This is a common issue in enrichment analysis: not all lipids within a subclass are involved in the disease, so the signals from the few important lipids can be lost when combined with many unrelated lipids in the same class.

Our lipidomic findings align with and extend prior studies of fatty liver and atherosclerosis. A metabolomics study of lean and obese metabolic-associated steatotic liver disease (MASLD) patients revealed that obese MASLD was characterized by increased levels of multiple long-chain phosphatidylcholine (PC) species, including PC (40:4)^34^. This aligns with our observation that PC 40:4 is implicated in MAFLD. Similarly, Shao et al. used mass spectrometry–based metabolomics to characterize nonobese and obese MAFLD patients with and without carotid atherosclerosis. They identified specific PCs and phosphatidylethanolamine species, including PCs (18:2/20:2), that were associated with the presence of carotid plaques^35^. PC (18:2/20:2) corresponds to PC (38:4), which shares similar total chain length and unsaturation with PC 40:4, suggesting possible metabolic parallels. PC (40:4) is found in various biological locations, such as blood, urine, feces, and cell membranes, and has been linked to several health conditions, including atherosclerosis, breast cancer, and ulcerative colitis^36–38^. These findings indicate that similar glycerophospholipid changes occur in both liver steatosis and atherosclerosis.

The identification of PC 40:4 specifically is intriguing from a biological standpoint. PC is the principal phospholipid of plasma lipoproteins and is essential for their assembly and secretion^39^. Impaired hepatic PC biosynthesis markedly reduces very-low-density lipoprotein (VLDL) secretion and lowers circulating VLDL and HDL levels. For example, the reduced activity of hepatic phosphatidylethanolamine N-methyltransferase (PEMT), an enzyme in the PC synthesis pathway, leads to defective VLDL particles and altered plasma lipid profiles^40^. Thus, dysregulation of PC metabolism can directly influence both liver fat content and blood lipoproteins. In addition, PC species can impact atherosclerosis; for example, certain PCs on high-density lipoprotein (HDL) promote cholesterol efflux from macrophages, a protective anti-atherogenic process^41^. Conversely, PCs are major components of low-density lipoprotein (LDL), and their oxidized forms can serve as damage-associated molecular patterns (DAMPs), activating inflammatory receptors and contributing to endothelial dysfunction and plaque formation^42,43^. Taken together, these findings underscore the importance of PCs in lipid homeostasis and suggest that shifts in specific PC species, such as PCs (40:4), could link hepatic steatosis with vascular inflammation.

As previously noted, PC 40:4 was quantified based on sum composition using scheduled MRM analysis, which does not distinguish structural isomers such as sn-position or acyl chain configurations. Therefore, the observed signal may reflect multiple PCs sharing the same sum composition (e.g., PC 18:0/22:4 or PC 20:0/20:4). This limitation is inherent in most targeted lipidomics workflows and should be considered when interpreting the specificity of the signal. Nevertheless, the consistent association of this lipid species across two distinct disease endpoints supports its relevance as a marker of shared metabolic disruption.

In addition to our data, other lipidomic studies of atherosclerotic plaques support a role for PCs in vascular disease. For example, imaging-mass spectrometry analyses have shown that PCs are enriched in macrophage-rich (vulnerable) regions of symptomatic carotid plaques, whereas more stable plaques often exhibit distinct phospholipid profiles, such as relatively high levels of lysophosphatidylcholine and cholesteryl esters^44^. Our results, which highlight a common PC species in both liver and plaque pathology, are consistent with the idea that systemic lipid remodeling underlies both MAFLD and CAP.

Although our findings are based on a Finnish cohort, the biological roles of phosphatidylcholine species—including PC 40:4—in lipid metabolism, lipoprotein transport, and vascular inflammation are fundamental processes that are conserved across human populations. However, lipidomic profiles are shaped by both interindividual factors (e.g., genetic background, diet, lifestyle) and time-sensitive physiological states (e.g., hormonal fluctuations, inflammation, or recent behavioral changes). For example, at least 18 lipid species have been shown to fluctuate across the menstrual cycle, and acute dietary or physical activity challenges can alter levels of circulating TAGs and sphingolipids^45–47^. The consistency of PC 40:4 as a marker across both MAFLD and CAP, despite this lengthy time interval, suggests that this lipid species captures a fundamental and stable aspect of cardiometabolic dysregulation rather than transient metabolic fluctuations. Future research in diverse, multiethnic cohorts will also be essential to determine its generalizability as a cardiometabolic biomarker.

A key limitation is the 11-year interval between lipidomic profiling (2007) and disease outcome assessment (2018), which complicates temporal interpretation. Earlier YFS data revealed that some participants classified as cases in 2018 already showed signs of MAFLD (in 2011) or CAP (in 2007), suggesting that the case groups may include both incident and prevalent disease. As such, the observed lipid associations could reflect either causal precursors or early disease-related changes. While PC 40:4 emerged as a consistent marker across both diseases, this observational design cannot determine whether its role is causal or consequential. Future longitudinal or mechanistic studies are needed to clarify the temporal dynamics and functional relevance of this lipid species.

Beyond the methodological considerations discussed above, this study also has other limitations. The sensitivity of the lipidomics platform, the possibility of residual confounding, and preprocessing steps may influence the results. Additionally, because the cohort consists of Finnish adults, the generalizability of our findings to more diverse or multiethnic populations may be limited. Future replication in other demographic groups is warranted.

In conclusion, our findings provide preliminary evidence that PC 40:4 may represent a shared lipidomic signature of MAFLD and CAP, despite their distinct clinical presentations. This overlap could indicate common underlying metabolic disturbances, such as dysregulated phosphatidylcholine metabolism or lipoprotein remodeling. If validated, PC 40:4 might serve as a candidate biomarker for identifying individuals at heightened risk for both hepatic and vascular complications. However, as this is an observational study based on associations, future longitudinal and mechanistic studies are needed to determine whether PC 40:4 plays a causal role in disease development or reflects a downstream effect. In addition, it remains to be clarified how modifiable lifestyle factors—such as diet, physical activity, or pharmacologic interventions—affect levels of PC 40:4 and whether they could be leveraged to reduce cardiometabolic risk.

## Supplementary Information

Below is the link to the electronic supplementary material.

Supplementary Material 1

Supplementary Material 2

## Data Availability

The dataset supporting the conclusions of this study was obtained from the Cardiovascular Risk in Young Finns study, which comprises health-related participant data. The use of data is restricted under the regulations on professional secrecy (Act on the Openness of Government Activities, 612/1999) and sensitive personal data (Personal Data Act, 523/1999, implementing the EU data protection directive 95/46/EC). Owing to these restrictions, the data cannot be stored in public repositories or otherwise made publicly available. Data access may be permitted on a case-by-case basis upon request only. Data sharing outside the group is performed in collaboration with the YFS group and requires a data-sharing agreement. Investigators can submit an expression of interest to the chairperson of the publication committee, Prof. Olli Raitakari (University of Turku, Finland), Prof. Mika Kähönen (Tampere University, Finland) and Prof. Terho Lehtimäki (Tampere University, Finland). Requests to access these datasets should be directed to OR, [olli.raitakari@utu.fi]; TL, [terho.lehtimaki@tuni.fi]; MK, [mika.kahonen@tuni.fi].
