# Supplementary material for: Uncovering the similarities of lipidome-wide markers of carotid artery plaque and metabolic dysfunction-associated fatty liver disease: the Young Finns study
Source: Sci Rep. 2026 May 6;16:20810. doi: 10.1038/s41598-026-51430-0 (PMC13338269; doi:10.1038/s41598-026-51430-0)
Supplement: Supplementary file 1 — Supplementary Material 1 [file 41598_2026_51430_MOESM1_ESM.docx]

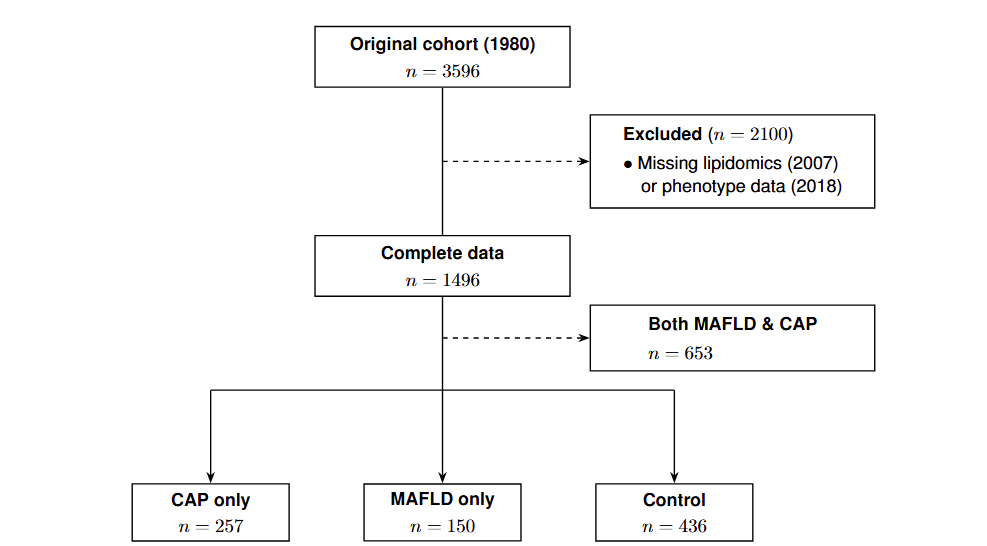


**Figure S1**. Out of 3,596 participants in the original 1980 cohort, 1,496 had complete lipidomic data from 2007 and phenotype data from 2018 and were included in the analysis. A total of 2,100 participants were excluded due to missing lipidomic or phenotype data. Among those with complete data, 653 participants presented with both MAFLD and CAP and were excluded from the primary 3-group comparisons to maintain mutually exclusive disease groups. The final analysis included three non-overlapping groups: participants with CAP only (n = 257), MAFLD only (n = 150), and controls with neither condition (n = 436).


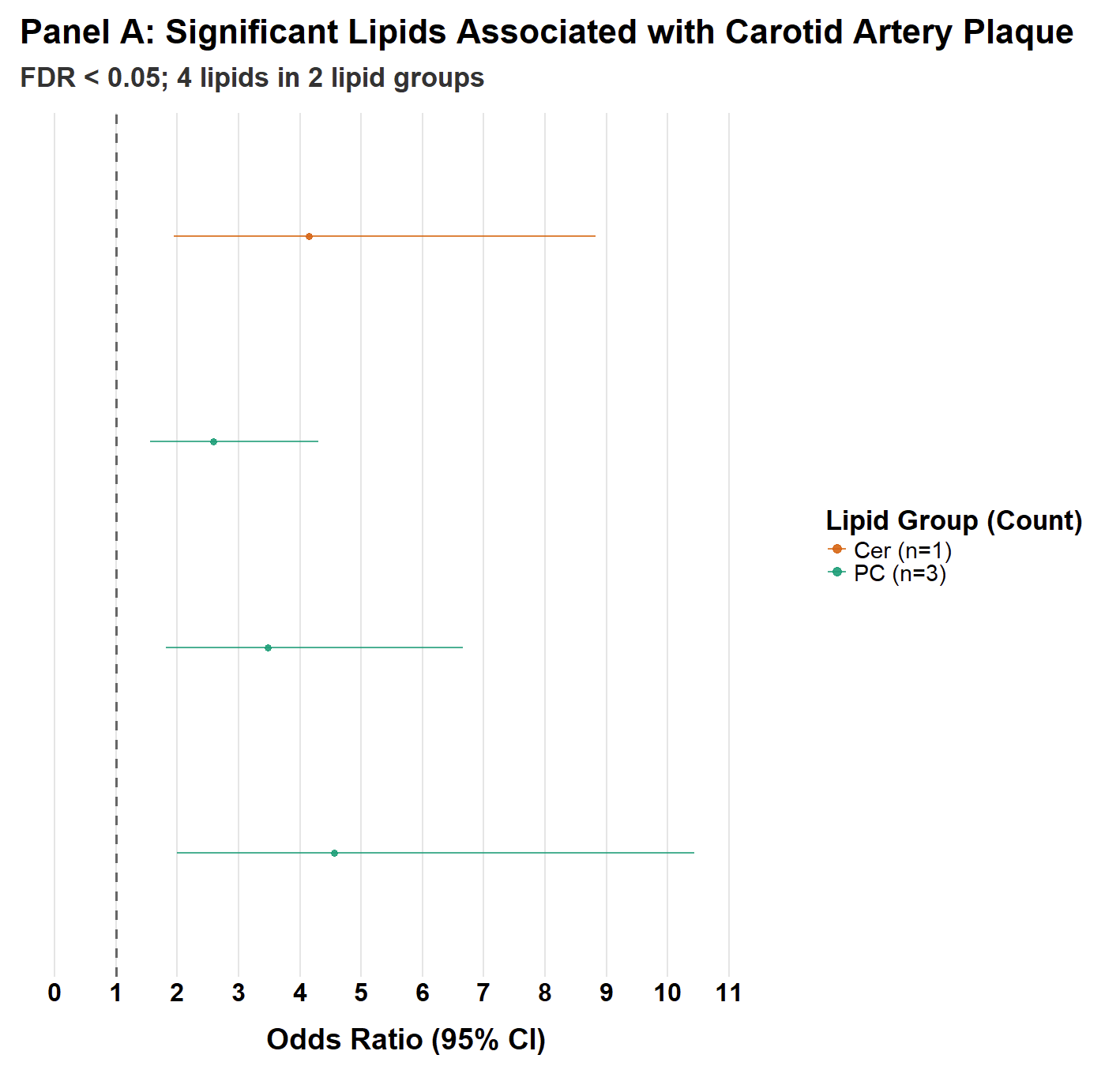

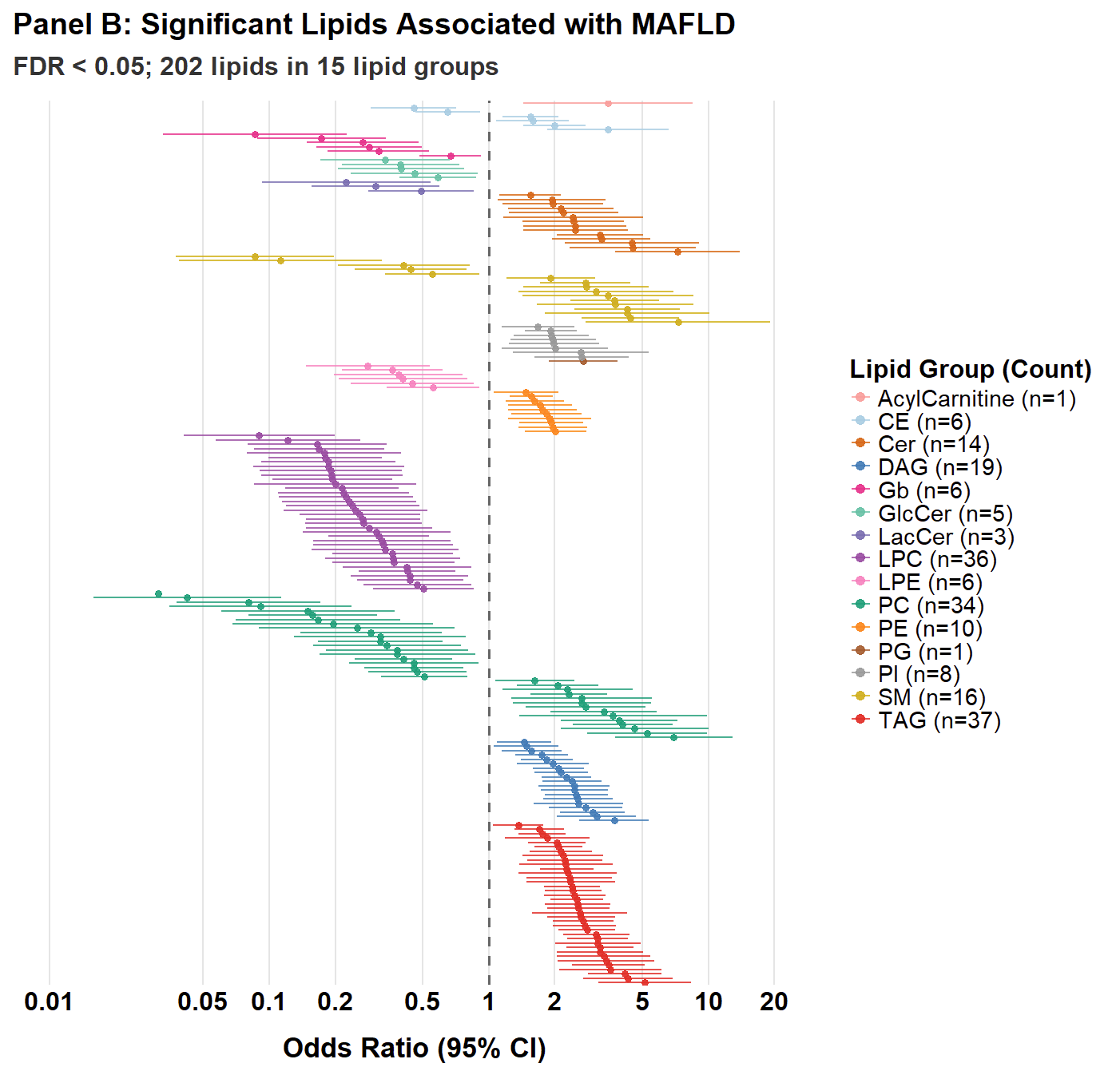


**Figure S2** . Lipids significantly associated with MAFLD and CAP in the adjusted model. The panel A shows odds ratios (OR) and 95% confidence intervals (CI) and all significant associations with CAP, while the panel B shows corresbondingly significant associations with MAFLD.


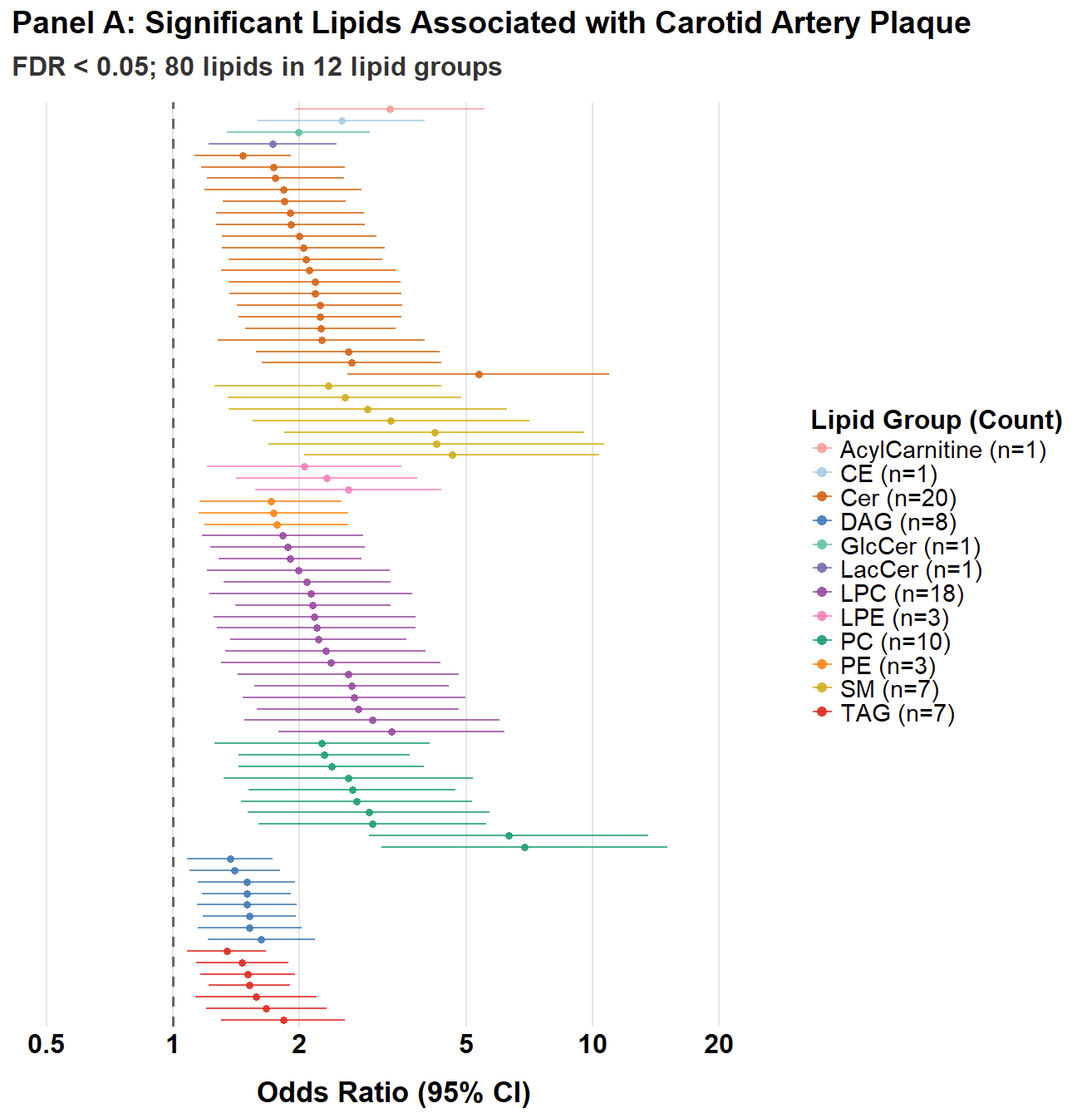


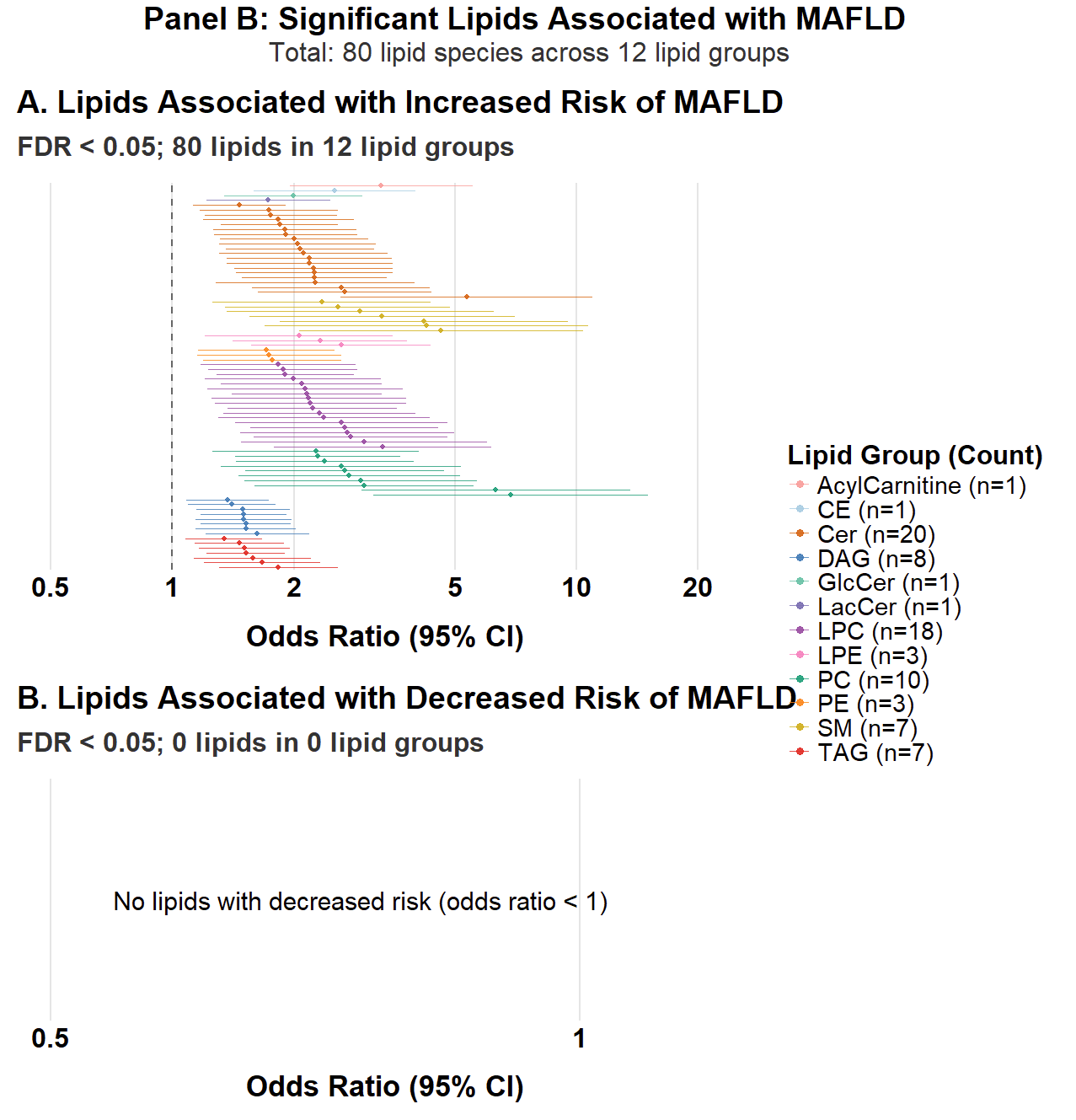


**Figure S3**. Shows odds ratios (OR) and 95% confidence intervals (CI) and significant lipids associated with CAP in unadjusted models. The panel A, displays all significant associations, while the panel B divides them into lipids associated with increased and decreased risk with CAP.


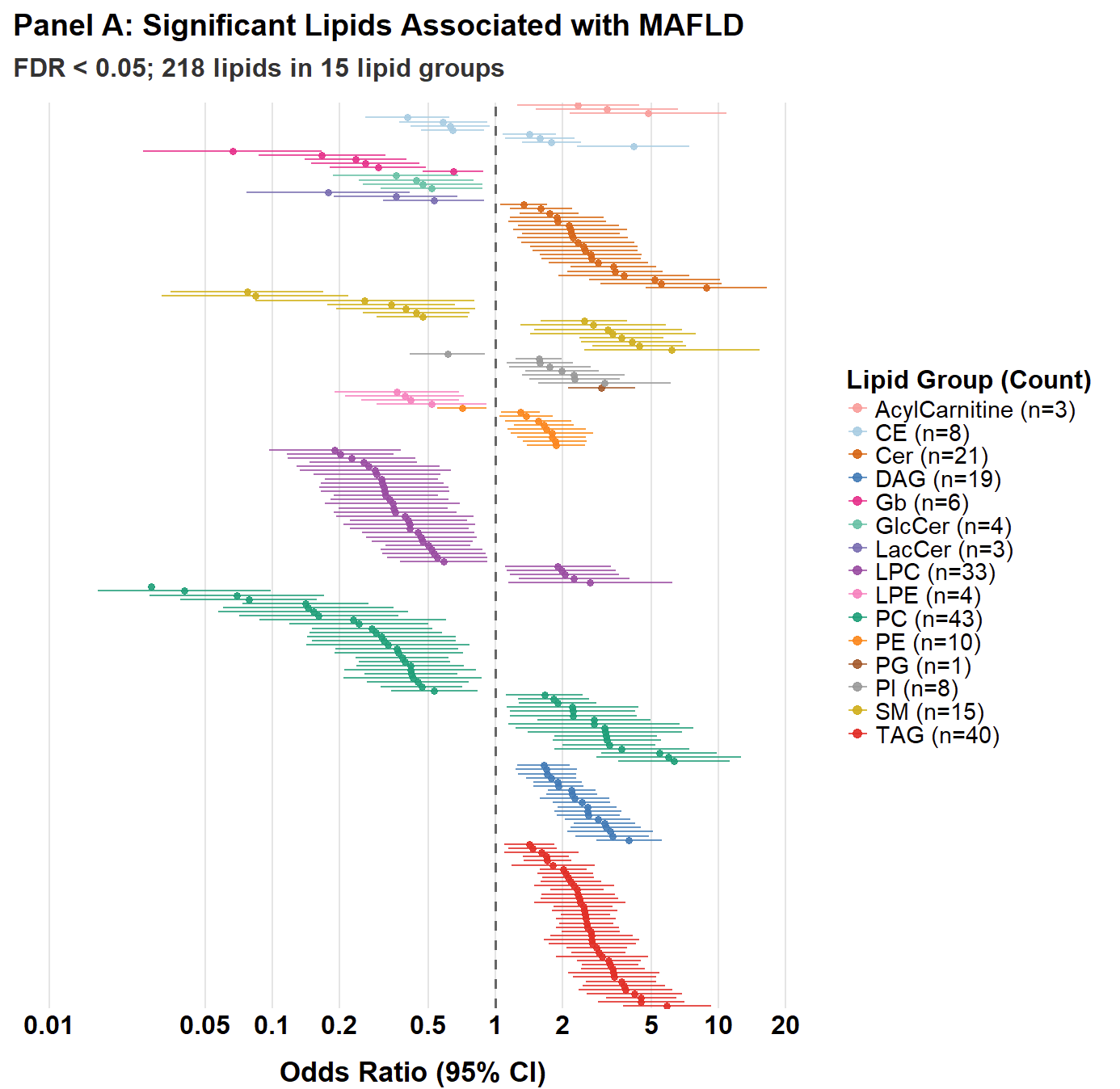


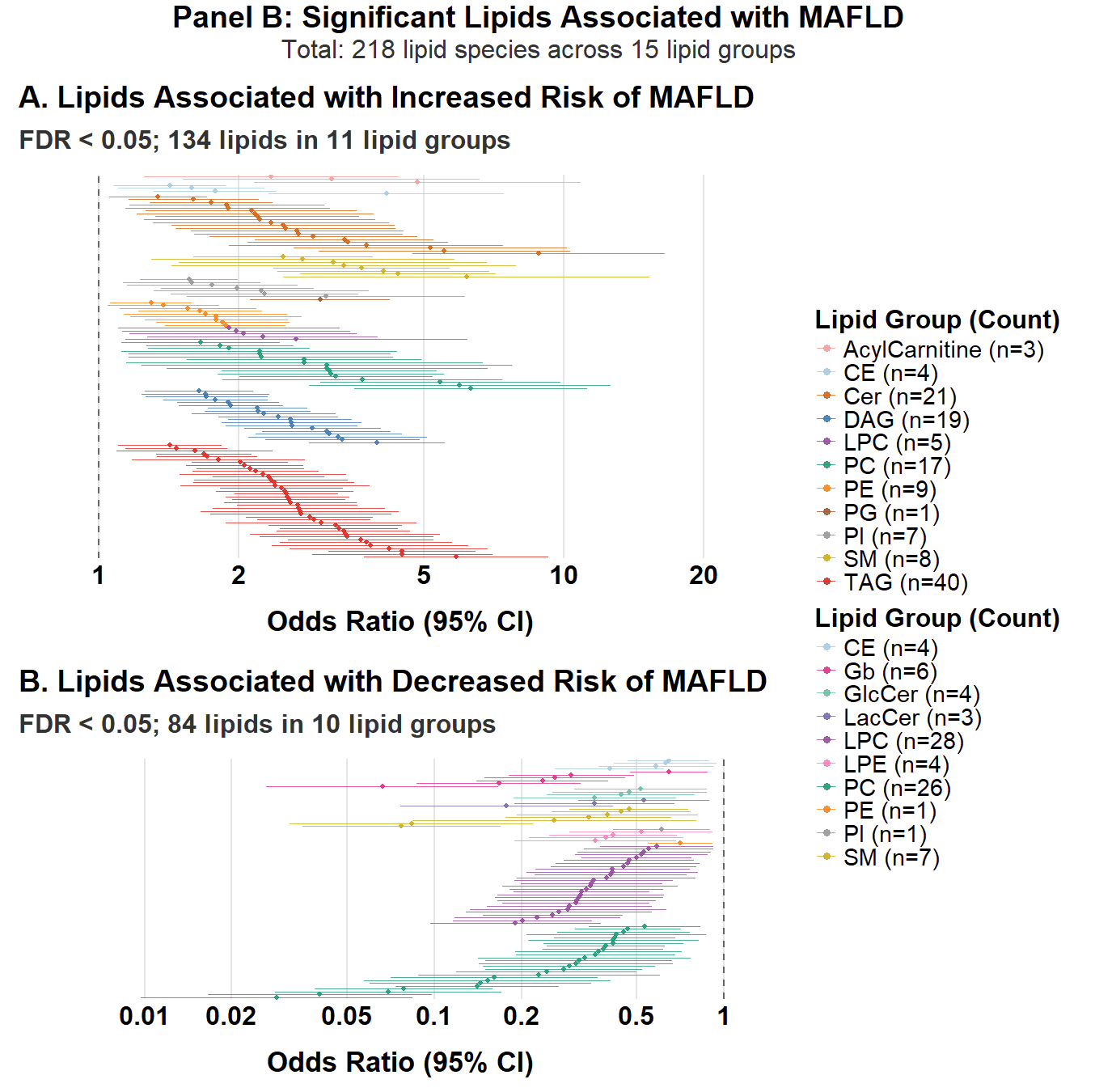


**Figure S4**. Shows odds ratios (OR) and 95% confidence intervals (CI) and significant lipids associated with MAFLD in unadjusted (crude) models. The panel A shows all significant associations, while the panel B separates lipids into those associated with increased (A) and decreased risk with MAFLD (B).


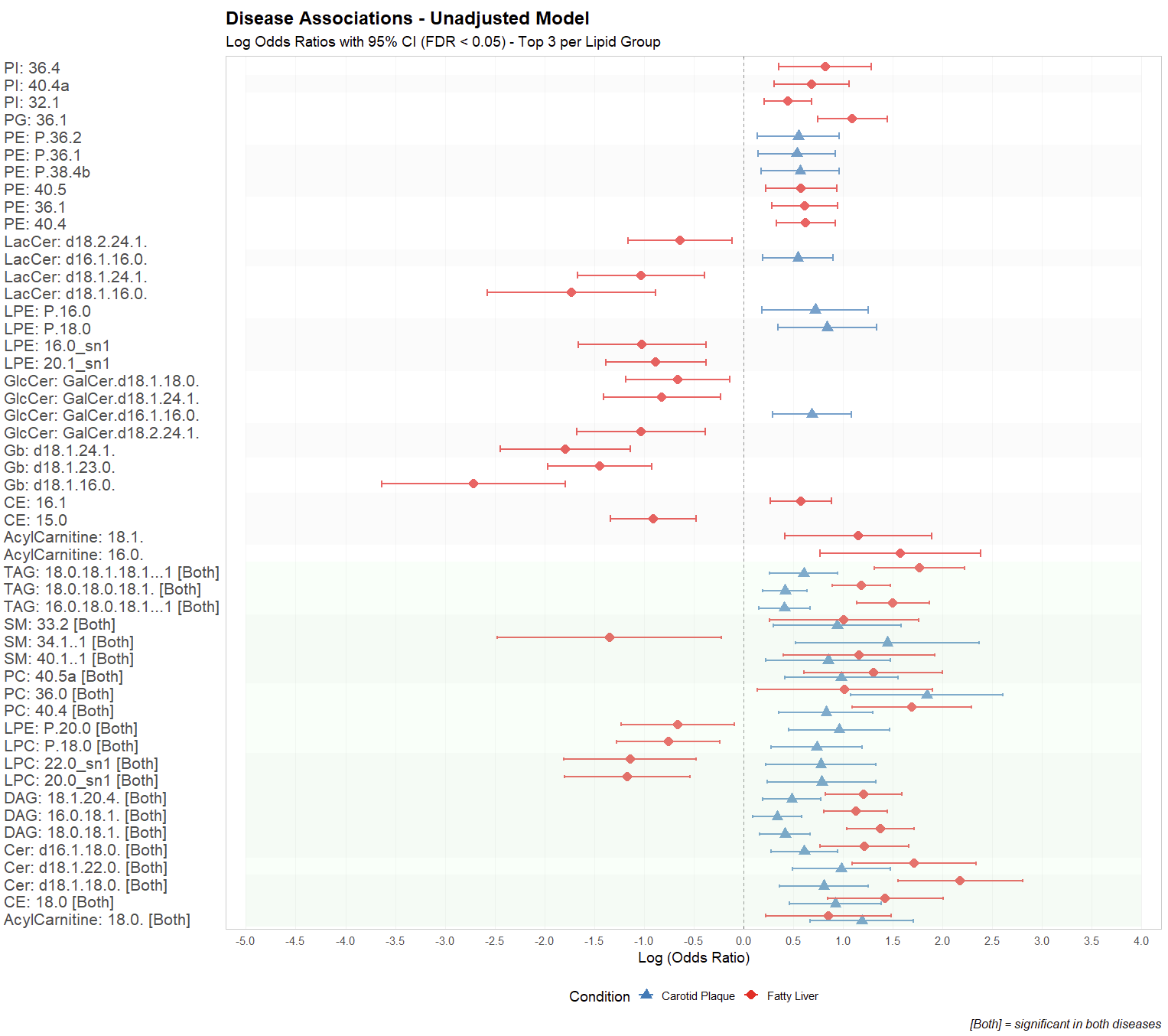


**Figure S5.** Forest plot illustrates the associations between lipid species and two diseases: CAP and MAFLD for the unadjusted model. Results are expressed as log odds ratios with 95% confidence intervals (CI) for lipids significantly associated with each disease (FDR < 0.05). This figure represents the unadjusted model. The top 3 lipids per lipid group are highlighted based on the minimum FDRs. In addition, the most significant overlapping lipids per class (based on FDR) are included to highlight shared associations between the two diseases, and these overlapping lipids are marked as "[Both]". Red points and lines represent associations with fatty liver, blue points and lines represent associations with carotid plaque, and green shading highlights lipids significant in both diseases.

Note: Due to the wide range of odds ratios across lipid species, log-transformed odds ratios are used to symmetrize the scale around zero, simplifying the visualization and comparison of both direction and magnitude of associations.


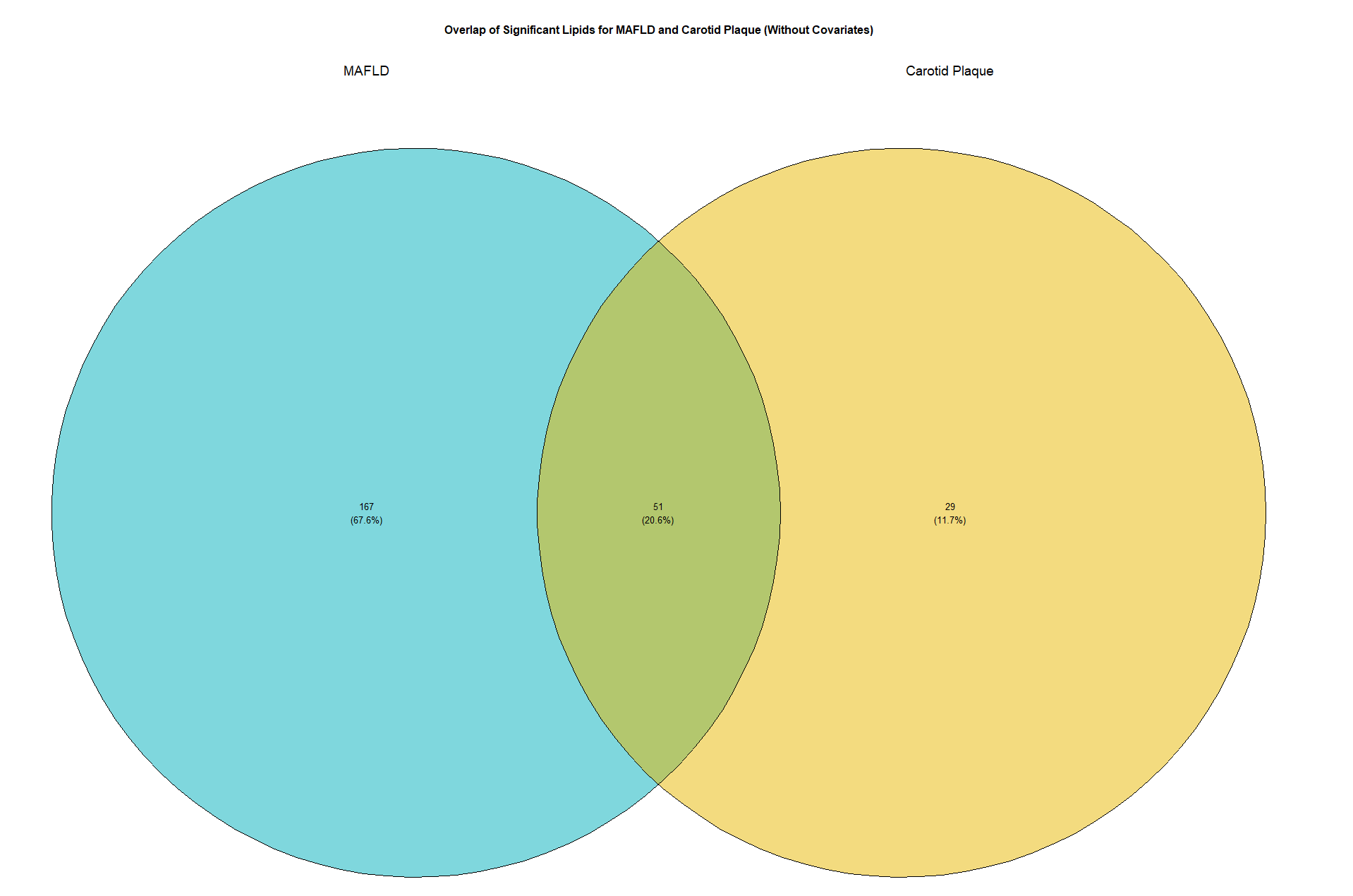


**Figure S6.** Overlap (green) of significant lipids between MAFLD (blue) and CAP groups (yellow) in unadjusted (crude) models.
